# Morphological Background Detection and Illumination Normalization of Text Image with Poor Lighting

**DOI:** 10.1371/journal.pone.0110991

**Published:** 2014-11-26

**Authors:** Guocheng Wang, Yiwen Wang, Hui Li, Xuanqi Chen, Haitao Lu, Yanpeng Ma, Chun Peng, Yijun Wang, Linyao Tang

**Affiliations:** 1 State Key Laboratory of Electronic Thin Films and Integrated Devices, School of Microelectronics and Solid-State electronics, University of Electronic Science and Technology of China, Sichuan, China; 2 Electrical and Computer Engineering, Kaiserslautern University of Technology, Kaiserslautern German Gottlieb-Daimler-Strabe, Kaiserslautern, Germany; Xiamen University, China

## Abstract

In this paper, some morphological transformations are used to detect the unevenly illuminated background of text images characterized by poor lighting, and to acquire illumination normalized result. Based on morphologic Top-Hat transform, the uneven illumination normalization algorithm has been carried out, and typically verified by three procedures. The first procedure employs the information from opening based Top-Hat operator, which is a classical method. In order to optimize and perfect the classical Top-Hat transform, the second procedure, featuring the definition of multi direction illumination notion, utilizes opening by reconstruction and closing by reconstruction based on multi direction structuring elements. Finally, multi direction images are merged to the final even illumination image. The performance of the proposed algorithm is illustrated and verified through the processing of different ideal synthetic and camera collected images, with backgrounds characterized by poor lighting conditions.

## Introduction

With the increasing development of digital image capturing devices, such as digital cameras, mobile phones and PDAs, their resolution is almost high enough to replace flatbed scanners. As a result, optical character recognition (OCR) techniques and content-based image analysis techniques are receiving intensive attentions in recent years and text-mining tools are becoming essential [1]. Among all the contents in images, text information has inspired great interests because of its high understandability by both human and computer, and wide applications, such as license plate reading, sign detection and translation, mobile text recognition, content-based web image search, object recognition, human computer interaction and so on [2], [3], [4]. In most cases, there is no guarantee to keep the environment in ideal condition during image capturing. In [5], an integrated image text information extraction system called TIE was described, but the system will be affected by noise pollution and uneven illumination [6], which is fatal.

Therefore, de-noising and illumination normalization are necessary in order to get higher performance. Some excellent performance de-noising algorithms, such as what's shown in [7], [8] and [9], [10], have been proposed, while non-uniform illumination is still a challenge in the field of text image recognition.

One of the most common techniques in normalization illumination image processing is histogram equalization and histogram specification, which are based on data statistical analysis, including global and local methods [11]. However, there are some disadvantages for the two methods. For the main disadvantage of the global method, the global properties of the image cannot be properly applied in a local context [12]; For the local method, it is easy to abate the image layers [13]. Then, based on the assumption that lighting modes of the image are known or can be estimated, Shan proposed a normalization method called quotient illumination relighting [14]. But in reality, it is difficult to get prior knowledge of an image, and it is impossible for an prior knowledge to be applied to all images. A method called homomorphic filter [15] performs well in image detail enhancement. However, as it works in the frequency domain, it takes a lot of effort in the transformation between time domain and frequency domain, yet considering poor local context. Another algorithm, proposed by Jimenez-Sanchez, divides image to blocks and takes the average of the max and minimal values of each blocks as the background of the corresponding areas [16]. This method will lead to blocking effect and fake contour. Hu provided a comparative study of 12 representative illumination preprocessing methods, but was limited to face recognition [17]. A local spatial co-occurrence based background modeling approach was presented to automatically estimate the local context background, but only worked well on human tracking [18], [19]. Xu proposed an algorithm using large-scale elements to implement Top-Hat transformation on images under varied lighting conditions [20]. This algorithm is simple and being widely used. However, due to the use of single structuring element, it cannot acquire good processing results under complex lighting conditions, and this method will result in blocking effect as well. Therefore, even though the reported algorithms to compensate changes in lighting varied, some are more adequate than others.

In this work, a morphological methodology to compute the image illumination background is proposed. Due to the nice characteristics of opening by reconstruction, which introduces almost no shape noise in both filtering and detection of structures [21], we replace the opening operation with it to develop the classical Top-Hat transform and further avoid blocking effect. Afterwards, modified Top-Hat operations with multi direction structuring elements (M-SEs) are applied to extract image information with different texture directions, components of which are fused to get a complete equalization illumination image.

The proposals given in this paper are illustrated with several examples. The proposed method and some other reported methods are compared, under the ideal synthetic images with four different lighting modes and captured images with noise characterized by 3 different languages texture.

This paper is organized as follows. Section 2 presents a brief background on some basic morphological operations. Section 3 illustrates a modified Top-Hat transform through the method of opening by reconstruction based on single structuring element(S-SE) to give an approximation to the background. In Section 4, the multi direction structuring elements (M-SEs) based illumination normalization algorithm in conjunction with entropy based image fusion is introduced. Section 5 shows a comparison among several other techniques. Finally, conclusions are presented in Section 6.

## Morphological Transforms

Mathematical morphology (MM), namely a collection of operators, is based on set theory and defined on an abstract structure. The abstract structure is an infinite lattice, which was first systematically examined by Matheron and Serra in the 1960s and is an extension of Minkowski's set theory [22]. For MM technology, its purpose is to analyze the shape and structures of the concerned target. Due to the powerful image analysis and image enhancement capabilities [23] with morphological transformation, including erosion, dilation, opening, closing, rank filters (including median filters), Top-Hat transforms, and other derived transforms, MM is widely used in the field of image processing and computer vision. MM involves structuring element set and image set.

### 2.1 Basis of MM transforms

MM involves image set and structuring element set (SE).

The result of erosion operation to an image shows where the SE fits the objects in the image. In gray scale, eroding an image 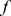 by SE 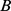 is defined as follows:
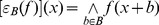
(1)


The result of dilation operation to an image shows where the SE hits the objects in the image. The dilation is expressed as follows:
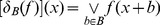
(2)


Opening (closing) is the sequential combination of erosion (dilation) and dilation (erosion) with SE and its reflection. The idea behind opening is to dilate an eroded image in order to recover the eroded image as much as possible. In contrast, the closing is to recover the dilated image.
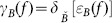
(3)

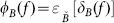
(4)


### 2.2 Opening and Closing by Reconstruction

Compared with opening and closing transforms, opening and closing by reconstruction are concepts that are more useful. They allow the elimination of undesirable regions without considerably affecting the remaining structures of the image, and will be helpful for recovering structures which are not completely destroyed by erosion or dilation. The characteristic arises from the way the transformation is built by means of geodesic dilation and erosion transform [24].

Geodesic dilation and erosion involve two images: mark image 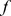 and mask image 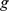. Mark image is the image to be processed; mask image plays a limited role in the spread of mark image's expansion.

Geodesic dilation and erosion of mark image relative to mask image are expressed as follows of one size scale, respectively
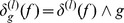
(5)

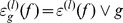
(6)


Here, 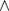 means taking minimum value point by point, and 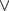 means taking maximum value point by point.

Geodesic dilation and erosion are dual operations.
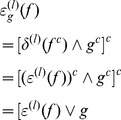



Of size 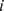, geodesic dilation and erosion of mark image 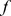 relative to mask image 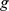 can be realized by iteration as:
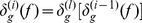
(7)

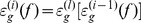
(8)


Conceptually this may continue indefinitely, but for all practical purposes iteration is terminated at an integer 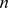 such that no change would occur after that.

The stable output termed as reconstruction by dilation and erosion is denoted by
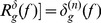
(9)

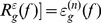
(10)


Here, 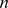 represents the iteration number, and reconstruction by geodesic dilation and erosion are dual operations

**Figure 1:**
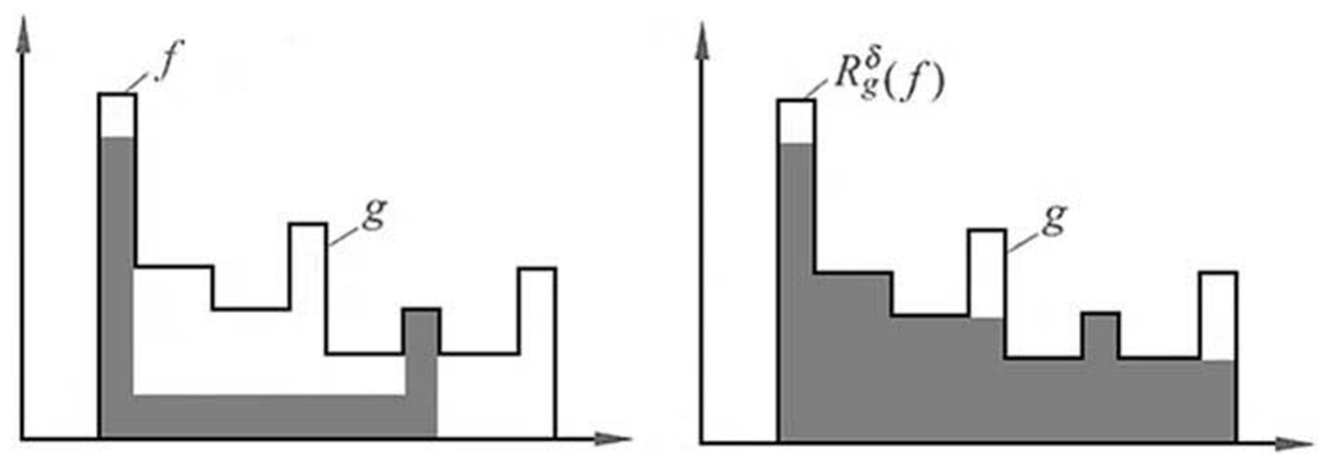
Illustrates reconstruction by opening of mark image 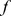 relative to mask image 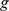.

**Figure 2:**
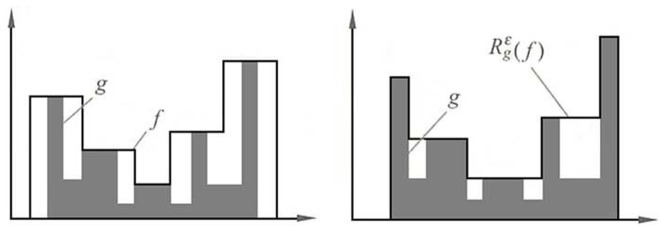
Illustrates reconstruction by closing of mark image 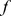 relative to mask image 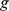.



When the mark image 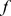 is equal to the erosion of the mask image 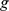, we will obtain the opening and closing by reconstruction as follow, as shown in Fig. 1 and Fig. 2.

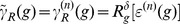
(11)

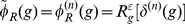
(12)


## The Modified Top-Hat Transform by Means of The Opening by Reconstruction

### 3.1 Classical Top-Hat transform

Usually, opening and closing operations are used in morphological filters to smooth the image. Opening an image will smooth the contours, eliminate small islands and sharp peaks or capes, while closing an image will smooth the contours, eliminates small holes and fills gaps on the contour.

The selection of proper morphological filter depends on the prior knowledge of target's sharp, size and direction. Opening and closing operations with SE will eliminate the structures unmatched with SE in image. These structures can be restored through difference operation between original image and its opening or closing results. Based on the difference operation, morphological transformations called WTH (white Top-Hat) and BTH (black Top-Hat) are proposed.

The WTH transformation obtains all bright features and sub graphs that are unable to accommodate SE:
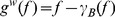
(13)


BTH is the dual operation of WTH, which sieves out the dark features and sub-graphs smaller than SE:
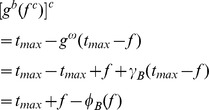



In practice, the BTH transform of image 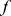 is defined as the difference between closing of the original image and the original image:
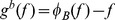
(14)


In text information images, information is represented by the intensity of transformation in image. The change of image information is more drastic and intensive than that of uneven illumination background, which means that the connected regions of image is much smaller than that of the illumination background in poor light.

Since opening (closing) operation can remove image features smaller than size of structural element SE, image area smaller than SE size will disappear after the opening function transformation, and connected regions bigger than the SE will be saved. Therefore, all features will be eliminated with brightness function retained if large scale SEs is used on image for opening (closing) operation.

Thus, the use of Top-Hat transform would output a uniform brightness image.

On the other hand, from the perspective of frequency domain filtering, Top-Hat transform works as a high-pass filter [25]. Since the image information locates in the high frequency domain with brightness gradient locating in the low frequency domain, the Top-Hat transform can be used for image light equalization.

**Figure 3:**
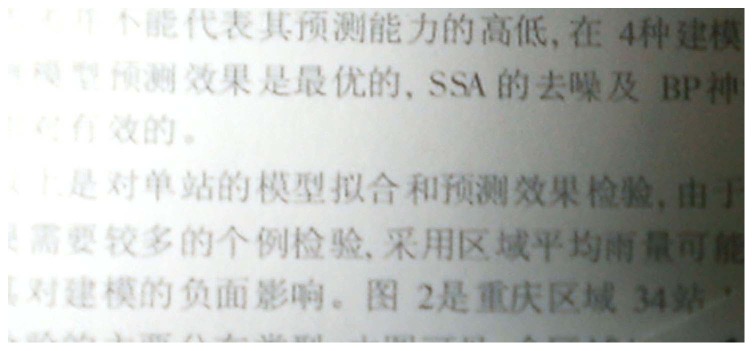
Uneven illumination text image.

**Figure 4:**
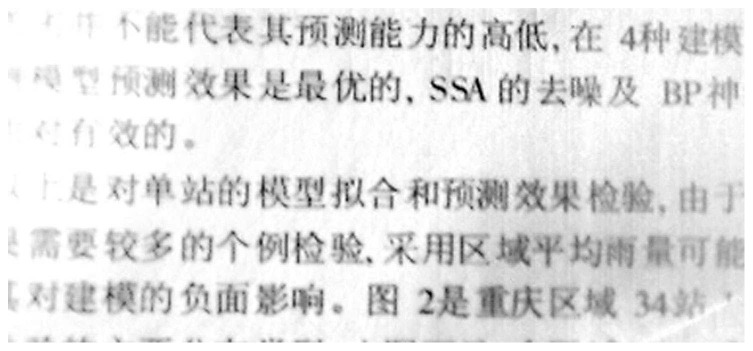
Result of Top-Hat transform with block effect.

As shown in Fig. 3 (the text is from [26]) and Fig. 4, an uneven illumination text image and result of Top-Hat transform is represented.

**Figure 5:**
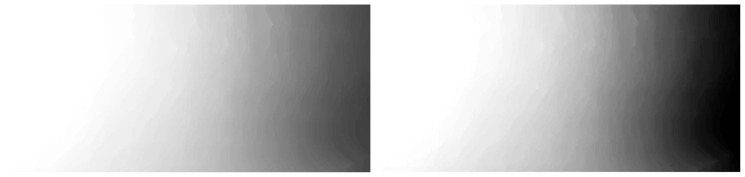
(a)–(b) Uneven background with block effect.

However, since the opening operation will generate new fake contours when the structuring element is increased, mutation at the edge of SE on extracted background brightness without a perfect preservation of the edge information [27] will occur. Therefore, edge noise will be introduced with opening based Top-Hat transform, and results in block effect. As shown in Fig. 4 and Fig. 5(a), which mean the illumination normalized result and the extracted uneven background with block effect. In order to clearly show the block effect, image gray-scale of Fig 5(a) is adjusted to a larger range, as shown in Fig 5(b). And some other images with obvious block effect are shown in Section 5.

### 3.2 Modified Top-Hat transform

**Figure 6:**
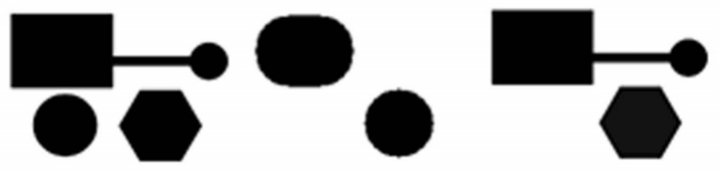
(a) Original image, (b) result of conventional opening of (a) using a disk SE, and (c) result of opening by reconstruction of (a) with same SE.

The main disadvantage of conventional opening and closing is that they do not allow a perfect preservation of the edge information and will introduce fake contours [28]. Banghamet al. [27] suggested an M-and N-sieves, which emphases on the size features, but ignores the shapes completely when solve the problem mentioned. However, it is possible to design morphological filters by reconstruction that satisfy the above requirement of preserving edge information and introducing no fake contours, and at the same time consider both shape and size features. Morphological opening by reconstruction [29] is such a filter. Compared with other morphology operations, opening by reconstruction can maintain patterns that were not removed completely by erosion. Fig. 6 shows the difference between conventional opening and opening by reconstruction.

Morphological opening by reconstruction is such a filter with characteristics of touching the regional minima and merging regional maxima [30], and it can be used to detect the background. Via replacing the opening operation with opening by reconstruction, a new WTH transform is proposed, which we call RWTH (Reconstruction Based WTH Transform) represented as follows
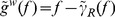
(15)


**Figure 7:**
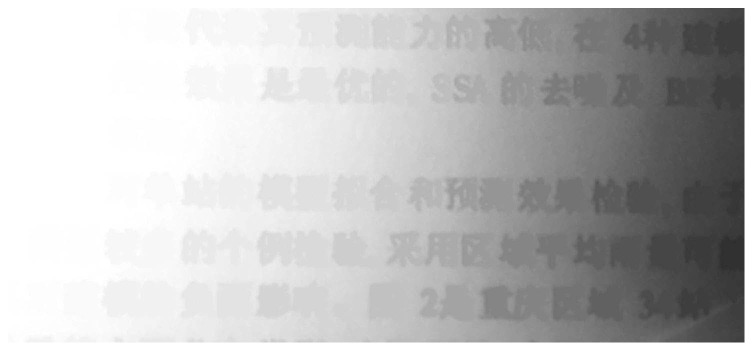
Background without block effect but with over illuminated.

Through RWTH transform, which is based on opening by reconstruction to equalize illumination, result shows balanced changes in the illumination background and no blocking effect, as shown in Fig. 7. Unfortunately, here arises another seemingly more serious problem, that part of the text information is mistaken as background illumination due to over-illumination equalization. Opening by reconstruction will reconstruct edge information of the features, leading to the spread of edge and part of the foreground image information being removed by RWTH transformation as background illumination.

In order to correct the over-illumination equalization phenomenon, closing by reconstruction, which is the dual operation of opening by reconstruction, is utilized to eliminate valid information mistaken as illumination background, and get real background estimation. The real background estimation is expressed as follows.
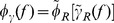
(16)


Thus, the real modified Top-Hat transform is obtained by replacing Eaq.15. In the following discussion, unless otherwise mentioned, "Top-Hat transform" refers to "the real modified Top-Hat transform", which is represented as follows.
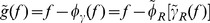
(17)


When these operators, i.e., sequential combination of closing by reconstruction and opening by reconstruction, are used, the problems above, such as blocking effect and over-illumination equalization, can be solved.

**Figure 8:**
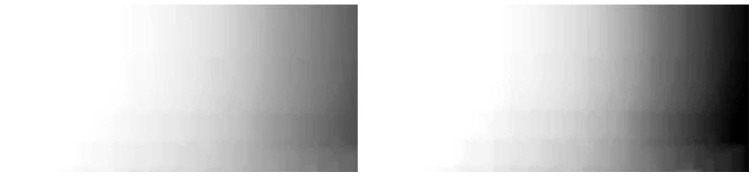
(a)–(b) Background without any block effect or over illumination.

The obtained illumination background is shown in Fig. 8(a). Fig. 8(a) is also adjusted in the same way as Fig. 5(a), and the adjusted image shown in Fig. 8(b) shows that the block effect is avoided effectually.

## The Multi Direction SEs Based Illumination Equalization Algorithm

### 4.1 Multi-direction SEs based top-hat transforms

When extracting the image features by Top-Hat transform, some simple and symmetrical shape structure elements, such as diamond, square and disk, are adopted. But these elements are sensitive to the image which has the same direction with SE, and thus edges with different directions from the SE will be smoothed. Therefore, using single SE to do background estimation cannot remain good image details, and will cause a big deal of background leakage especially when uneven lighting degree is large and image background is of strong fluctuation. When signal noise ratio is low, the gray value of background leakage would possibly be higher than the gray value, resulting in severe degeneration after image processing. Therefore multi-structure elements oriented in different directions [31] is used in Top-Hat transform for illumination normalization.

From the above analysis, it is obvious that Multi-direction SEs based top-hat transforms have more advantages over single structuring morphological transform. So we use big scale Multi-direction SEs to implement Top-Hat transform.

**Figure 9:**
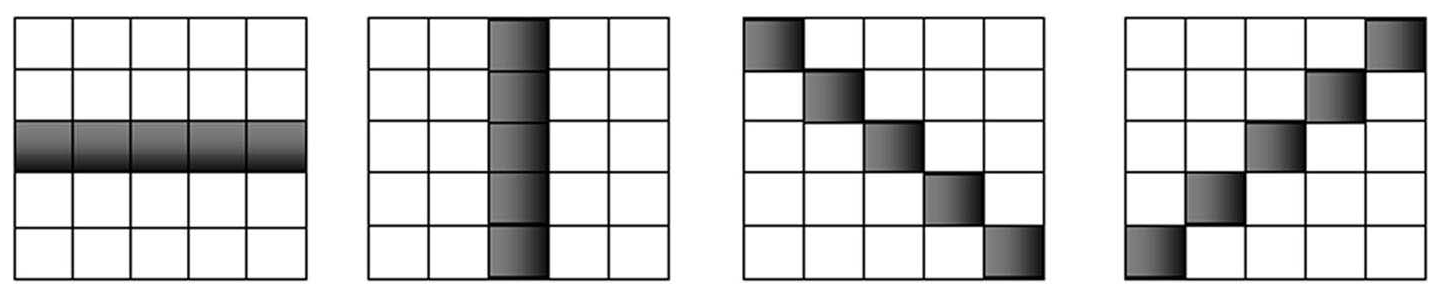
Different SEs used in proposed algorithm.

In this paper, based on Multi-direction SEs, uneven illumination background correction algorithm is proposed, and linear SEs with different directions 0°, 45°, 90° and 135° are selected, scales of which are 1/4 of the smallest scale of image row and column, as shown in Fig. 9.

### 4.2 Introduction to information entropy

Shannon proposed entropy in 1948, which is mainly used to measure the abundance degree of information. For single image, gray value of each pixel can be considered as independent, and entropy is defined as the amount of information contained in image, mathematically given as:
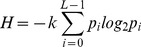
(18)


Here, 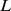 is the total number of grey levels, and 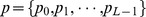 is the probability of occurrence of each level. Larger 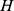 indicates more information.

For an 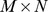 gray image, 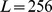, 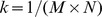, then entropy of the image is:
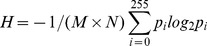
(19)


### 4.3 The illumination normalization algorithm based on Multi-direction SEs top-hat transforms

Information contained in text image is intuitively represented by the insensitive change of image. The change of text information presented by image is drastic, while change in uneven illumination background is relatively flat. Meanwhile, sub-images are obtained by extracting features of different directions through multi-direction SEs Top-Hat transform. Their information content can be measured by information entropy. Therefore, information entropy can be used as weights, as shown in the following, to merge the sub-graph, and to get the final illumination equalization image.
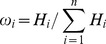
(20)


Here, 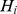 represents the entropy of image processed by Top-Hat transform with Multi-direction SEs, and 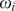 represents the image's weight factor, 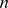 is the number of Multi-direction SEs.

From Equ.17 and Equ.20, we can get the final illumination equalization image, as shown in Equ.21.
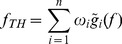
(21)


**Figure 10:**
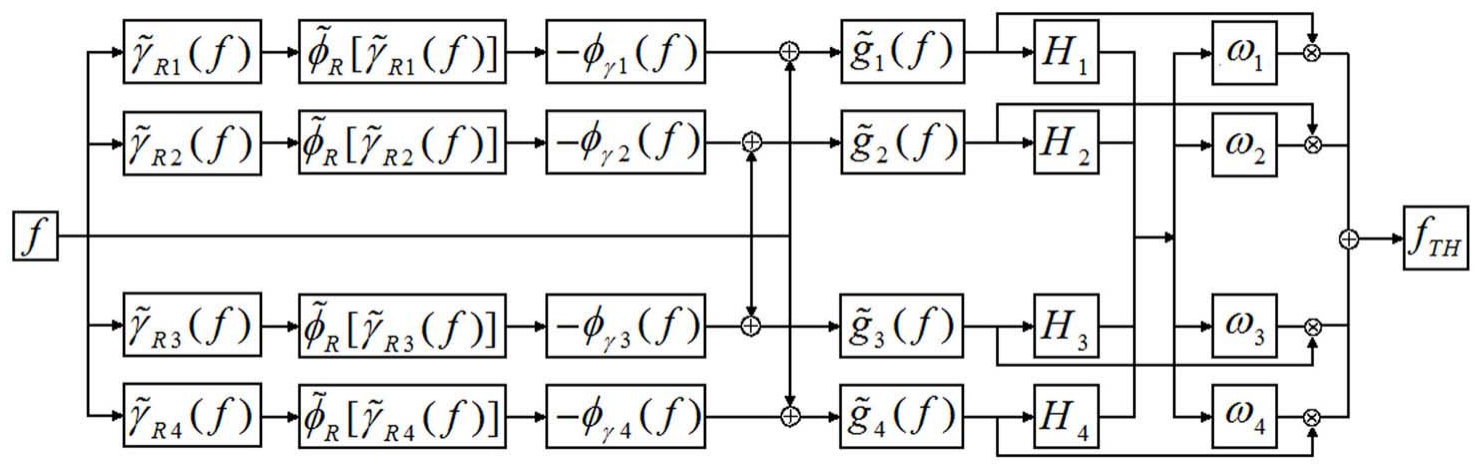
Diagram of the proposed algorithm.

The whole structuring diagram of the new algorithm based on multiple structures is illustrated in Fig. 10.

Then, another simple relation is employed to get a better image as shown in Equ.22.
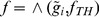
(22)


**Figure 11:**
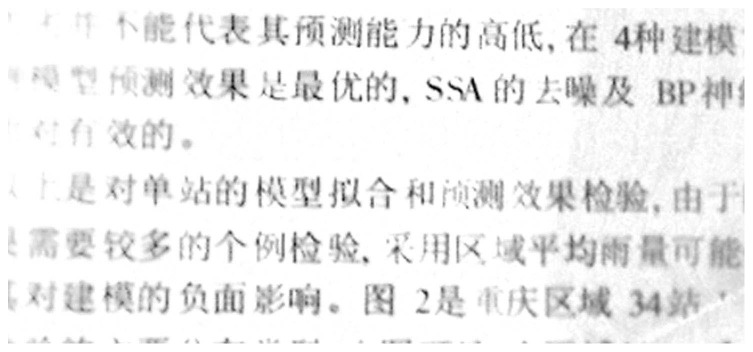
Final illumination normalized output without block effect or fake contours.

Finally, a simple contrast enhancement technology is used [16], and the final illumination normalized output of Fig. 3 is shown in Fig. 11.

## Some Other Tests and Discussions

**Figure 12:**
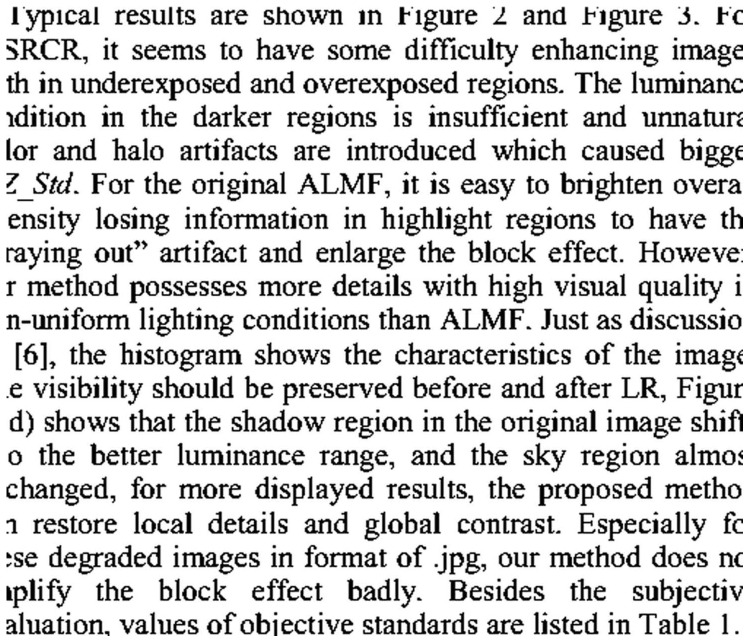
Ideal text image.

**Figure 13:**
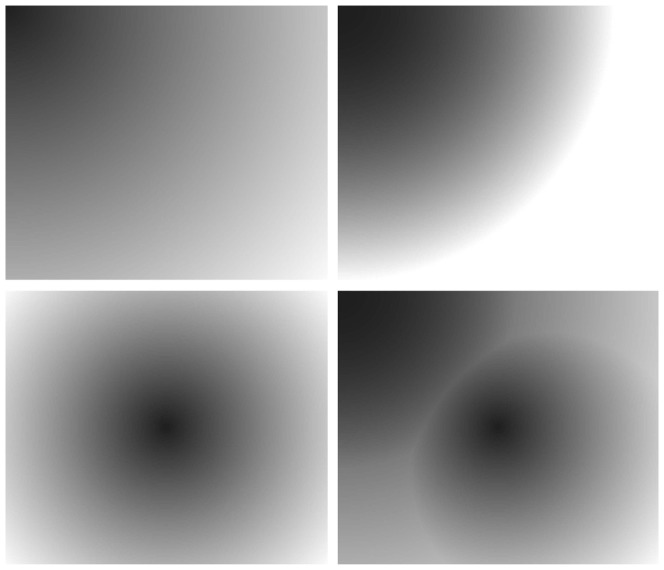
(a)–(d) Four different uneven illumination modes.

**Figure 14:**
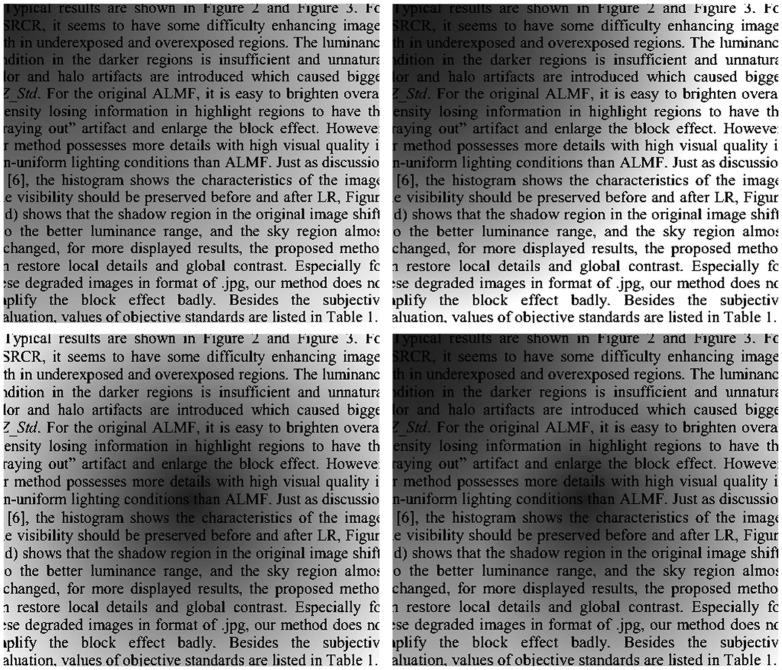
(a)–(d) Synthetic images with the four different uneven illumination modes.

The proposed algorithm has been applied on processing a set of images. Fig. 12 (the text is from [32]) is an ideal text image, and Figs. 13(a)–(d) show some different uneven illumination modes, with which synthetic images represented in Figs. 14(a)–(d) are generated to do tests in the experiment. The algorithm is also illustrated by some actual collected images with different textures characterized by Chinese, English and Japanese after the discussion of the ideal image.

Fig. 13(a) shows an uneven illumination of 1/4 circular. Image gray linearly increases from the top left corner to the lower right corner. Fig. 13(b) is based on Fig. 13(a), with more intense gray change. Fig. 13(c) presents a circular shaped uneven illumination, with linear gray scale increasing from the center to the edge. Uneven illumination of Fig. 13(d) is the most complex, which is a composite of the above three modes. The synthetic images are shown in Figs. 14(a)–(d).

**Figure 15:**
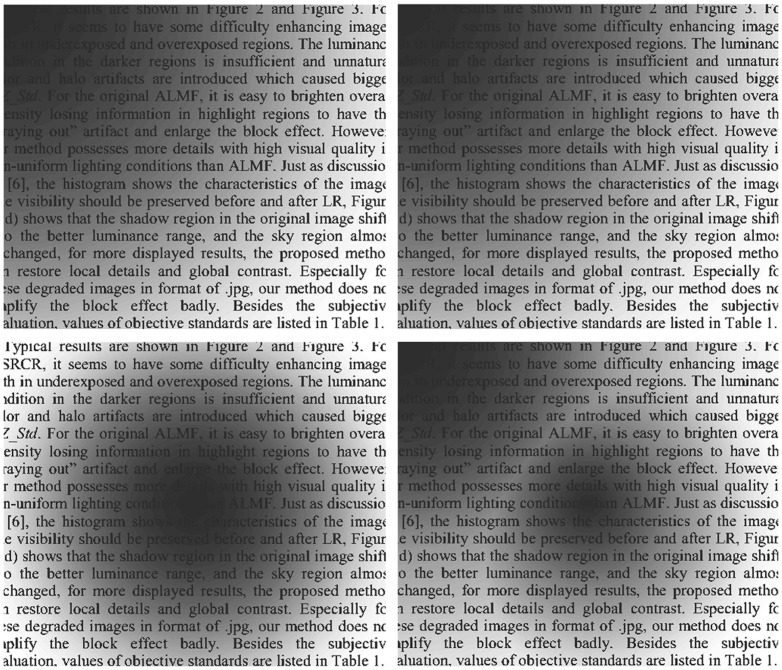
Results of histogram equalization algorithm.

**Figure 16:**
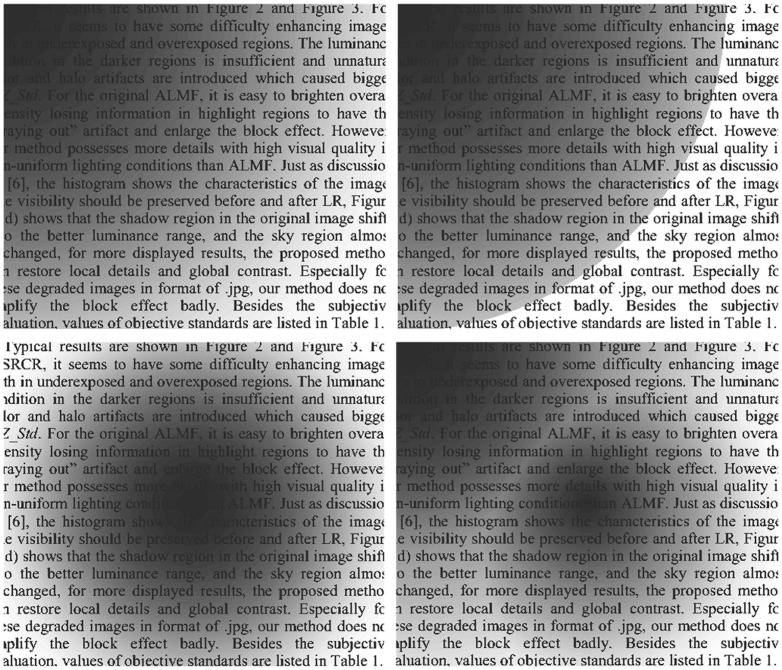
Results of histogram specification algorithm.

**Figure 17:**
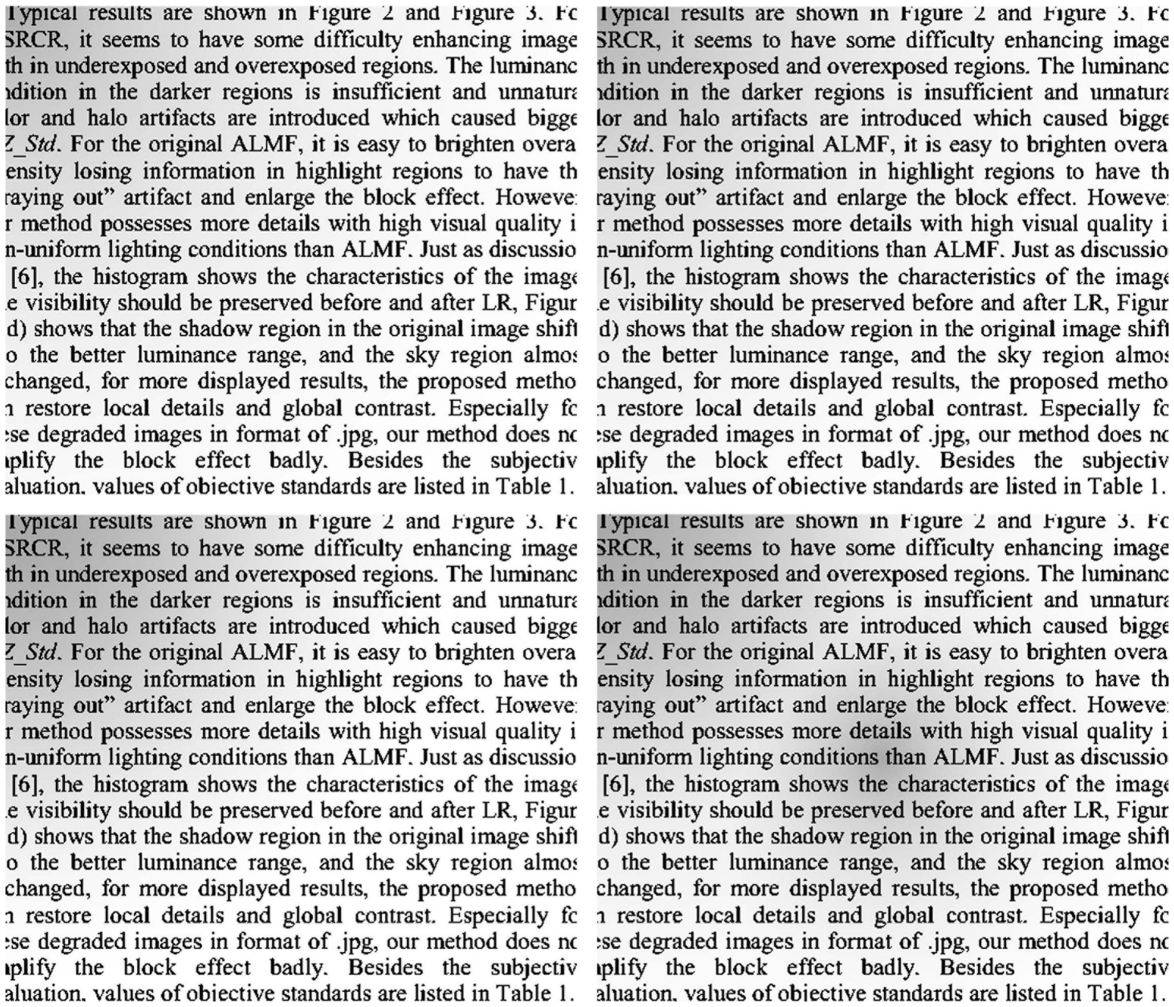
Results of method proposed by[16].

**Figure 18:**
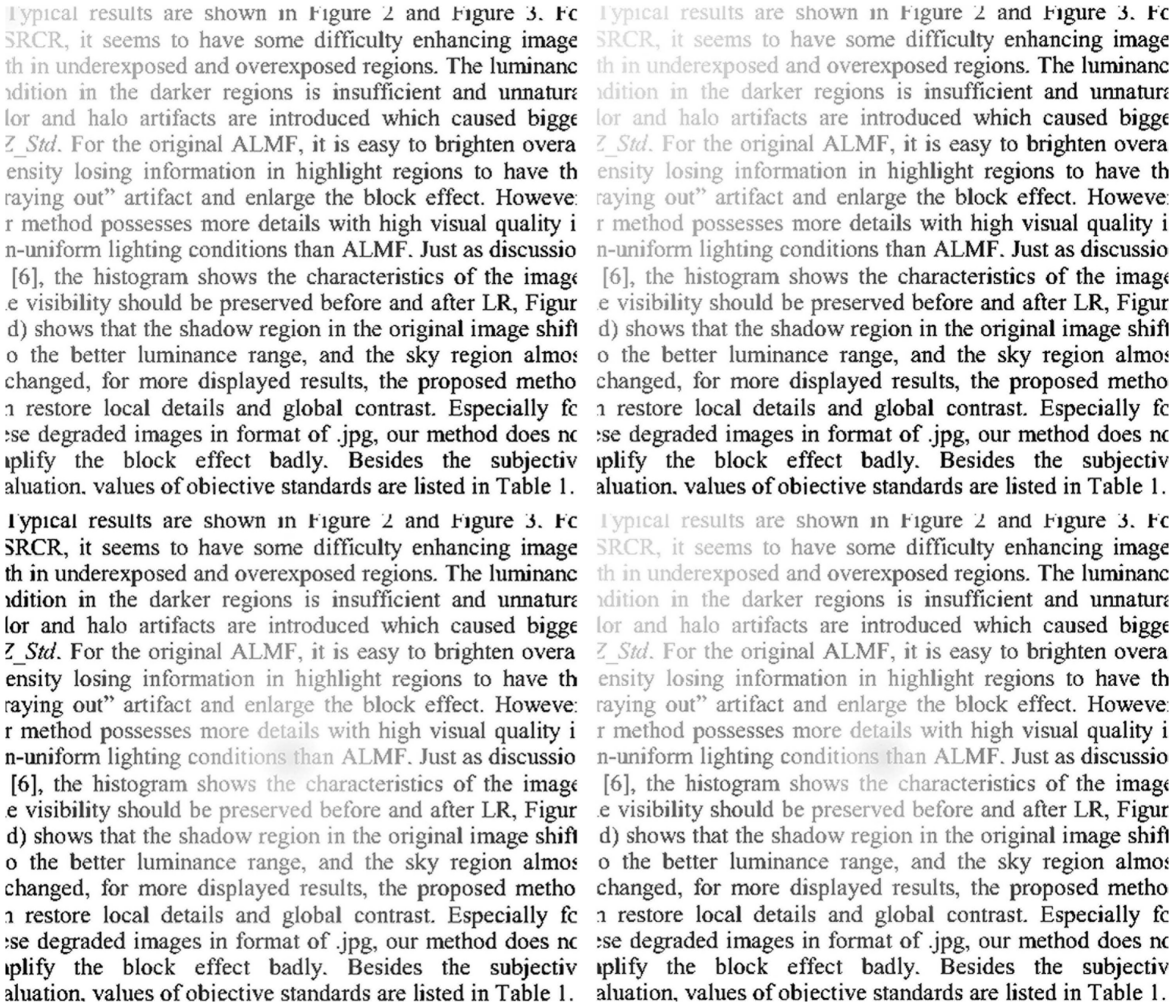
Results of classical TOP-HAT transform.

The results are compared with other three well-known methods. The results of histogram equalization and specification algorithm are shown in Figs. 15(a)–(d) and Figs. 16(a)–(d) in the identical order. Results show that contrast of the whole image is enhanced. Unfortunately there is no any improvement in the uneven illumination problem, even with new contours noise introduced. Figs. 17(a)–(d) show the respective results of method proposed by [16], combining opening by reconstruction with Weber's Law. This method preserves the text details well and enhances the image contrast, but without light uneven completely improved. Classical Top-Hat is a well done method, and performs well, shown in Figs. 18(a)–(d).

**Figure 19:**
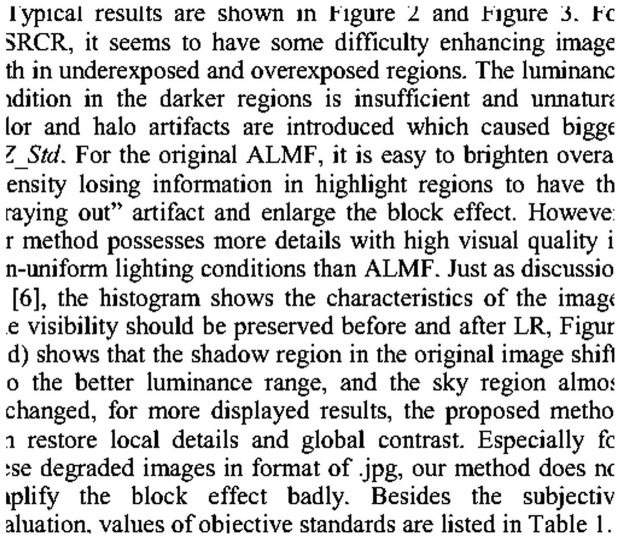
Results of the proposed algorithm.

For all the four different light modes in Fig. 14, results of the proposed algorithm are almost the same, so only one is shown in Fig. 19. Not only does it exhibit the best illumination normalization effect, but introduces no fake contours. The text details of images are also well retained.

It is necessary to mention the basic difference between the proposed method and the Classical Top-Hat transform, as they are conceptually most similar. The results of classical Top-Hat transform are shown in Figs. 18(a)–(d). The results are also nice, compared with the results of the proposed method, shown in Fig. 19. Actually, this is due to the low illumination variation frequency of synthetic images and ideal condition without noise introduced. Otherwise, because of the Top-Hat transform's inherent attribute by measuring large-scale connected area, classical Top-Hat transform will have serious block effects, and be sensitive to noise. When dealing with real images captured by digital devices, the proposed algorithm performs better than the classical Top-Hat transform.

**Figure 20:**
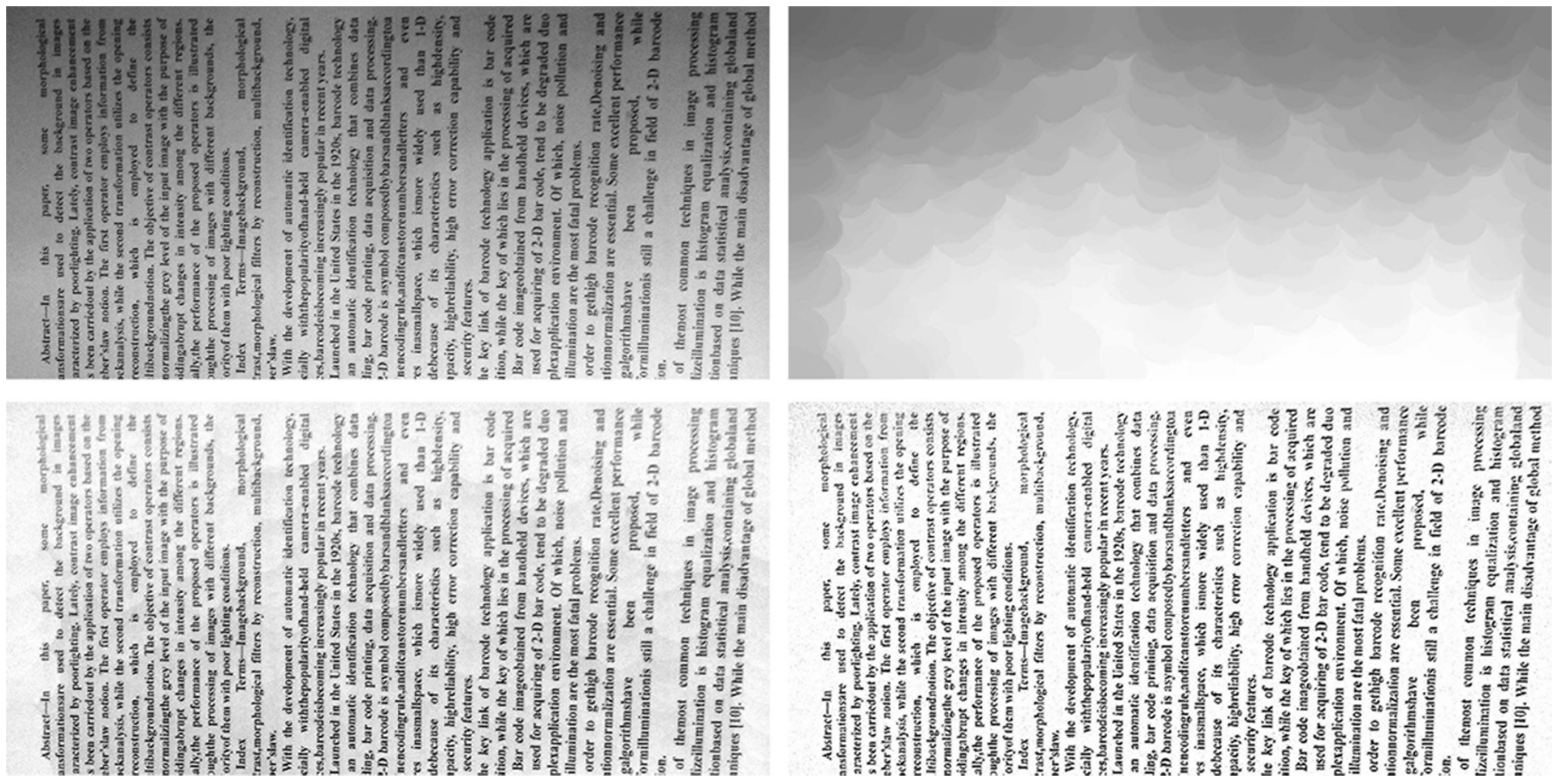
English text image, (b) uneven illumination background extracted by Top-Hat, (c) Equalization results from Top-Hat, (d) Equalization results from proposed algorithm.

**Figure 21:**
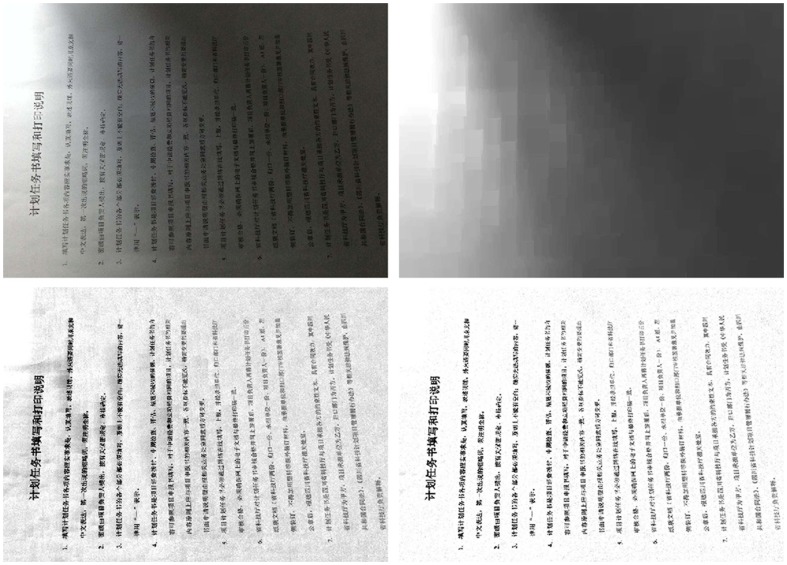
Chinese text image, (b) uneven illumination background extracted by Top-Hat, (c) Equalization results from Top-Hat, (d) Equalization results from proposed algorithm.

**Figure 22:**
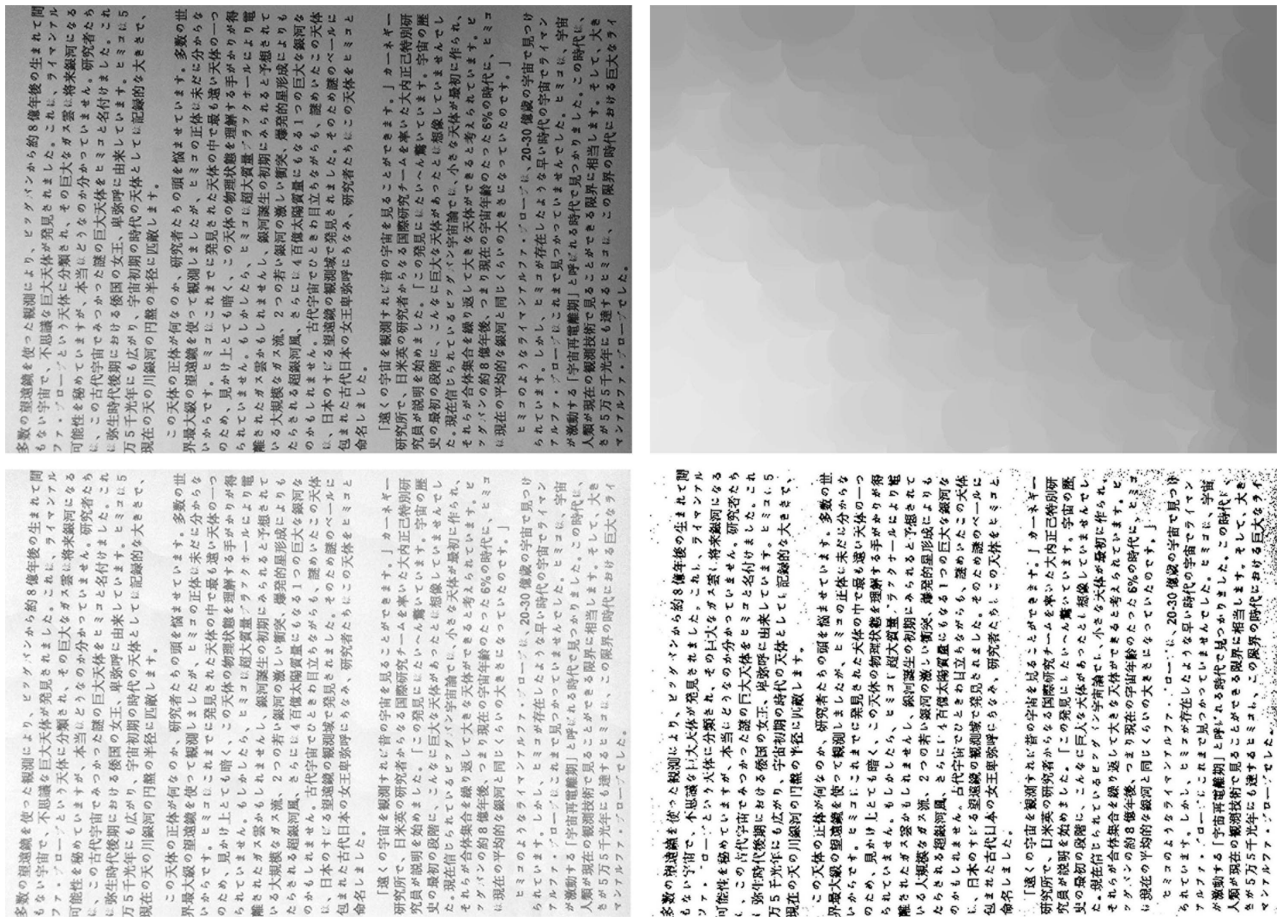
Japanese text image, (b) uneven illumination background extracted by Top-Hat, (c) Equalization results from Top-Hat, (d) Equalization results from proposed algorithm.

Some realistic images are collected to show comparison between the two algorithms. In order to illustrate applicability of the algorithm for different textures text image, Fig. 20(a) shows the captured English text image, Fig. 21(a) the Chinese text image, and Fig. 22(a) the Japanese text image. The Text of Fig. 20(a) is part content of this paper, the text of Fig. 21(a) is from [33], and the text of Fig. 22(a) is a notice on graduation design to obtain the bachelor's degree.

Conventional Top-Hat transform is performed on Figs. 20(a)–22(a). The extracted uneven illumination backgrounds are shown in Figs. 20(b)–22(b). The equalization results are shown in Figs. 20(c)–22(c). Results show that the uneven illumination has not been completely eliminated, and the extracted light backgrounds show block effect, especially in the case of noise introduced by capturing device. Figs. 20(d)–22(d) represent the final illumination equalization results obtained by employing the proposed algorithm on Figs. 20(a)–22(a). In addition, results show that this algorithm can efficiently act on images with poor lighting of different textures, and almost completely remove uneven illumination background which is smooth, as well as insensitive to noise introduced by capturing device.

In conclusion, the proposed algorithm can be used for uneven illumination text images with poor lighting. Algorithm works well on removing uneven illumination background without introducing new contours noise, and it is not sensitive to noise, and is suitable for images captured by digital devices which have high noise.

## Conclusions

This paper presents a study to detect the text image uneven illumination background and to normalize uneven illumination images with poor lighting. Firstly, a classical Top-Hat transform methodology, which would result in block effects and fake contour, was introduced to compute an approximation of the background. This transform was then redefined by replacing opening operation with opening by reconstruction, which is called RWTH transform. However, problem called over-illumination equalization was detected when the morphological opening by reconstruction was employed. Therefore, a dual operation called closing by reconstruction was introduced. Additionally, multi-direction structuring elements were used to modify the Top-Hat transform and balance the direction information.

The performance of the proposals was illustrated by several ideal examples with different uneven illumination modes, and some camera collected text images with Chinese, English and Japanese texture. In addition, the performance operators employed in this paper was compared with others given in the literature. Results show that the proposed algorithm is more advantageous over others in obtaining uniform illumination images, retaining edge information and introducing no contour. However, our proposed algorithm requires large amount of computation and storage space because of the loop definition of morphological reconstruction. Further studies will be carried out to optimize this algorithm to reduce its computation work and storage space, and implement this algorithm through ASIC (application specification integrated circuit).
